# Human Stem Cells and Articular Cartilage Regeneration

**DOI:** 10.3390/cells1040994

**Published:** 2012-11-05

**Authors:** Atsuyuki Inui, Takashi Iwakura, A. Hari Reddi

**Affiliations:** Center for Tissue Regeneration and Repair, Department of Orthopaedic Surgery, School of Medicine, University of California Davis, 4635 Second Avenue, Research Building I Room 2000, Sacramento 95817, CA, USA; Email: atsuyukiinui81@gamil.com (A.I.); t_iwakura1978@yahoo.co.jp (T.I.)

**Keywords:** stem cells, articular cartilage, growth factors

## Abstract

The regeneration of articular cartilage damaged due to trauma and posttraumatic osteoarthritis is an unmet medical need. Current approaches to regeneration and tissue engineering of articular cartilage include the use of chondrocytes, stem cells, scaffolds and signals, including morphogens and growth factors. Stem cells, as a source of cells for articular cartilage regeneration, are a critical factor for articular cartilage regeneration. This is because articular cartilage tissue has a low cell turnover and does not heal spontaneously. Adult stem cells have been isolated from various tissues, such as bone marrow, adipose, synovial tissue, muscle and periosteum. Signals of the transforming growth factor beta superfamily play critical roles in chondrogenesis. However, adult stem cells derived from various tissues tend to differ in their chondrogenic potential. Pluripotent stem cells have unlimited proliferative capacity compared to adult stem cells. Chondrogenesis from embryonic stem (ES) cells has been studied for more than a decade. However, establishment of ES cells requires embryos and leads to ethical issues for clinical applications. Induced pluripotent stem (iPS) cells are generated by cellular reprogramming of adult cells by transcription factors. Although iPS cells have chondrogenic potential, optimization, generation and differentiation toward articular chondrocytes are currently under intense investigation.

## 1. Introduction

Articular cartilage (AC) is an avascular tissue, which consists of abundant extracellular matrix. AC does not heal spontaneously under physiological circumstances due to innate avascularity, low cellularity and low cell turnover [1] that can lead to progression of osteoarthritis (OA). OA is the most common musculoskeletal disease in the elderly and could become the fourth leading cause of disability by the year 2020 [2]. So far, there are limited options for OA treatment, such as pharmaceutical interventions and joint replacement surgery [3]. In terms of AC regeneration, many procedures, such as subchondral drilling, abrasion arthroplasty, osteochondral grafts and transplantation of mesenchymal stem cells (MSCs) or chondrocytes, have been developed [4,5,6,7,8]. Differentiation toward cartilage was induced by morphogens, such as transforming growth factor (TGF)-β superfamily and members of the bone morphogenetic protein (BMP) family. The resulting differentiated chondrocytes expressed cartilage specific markers, such as type II collagen and Aggrecan. Currently, autologous chondrocyte transplantation is widely used for AC regeneration in clinic. However, donor site morbidity is a challenge [9]. Use of stem cells in combination with growth factors and scaffolds is another option for AC regeneration [1]. In the present review, we discuss chondrogenesis using stem cells and morphogenetic proteins for potential applications in regeneration of AC.

## 2. Stem Cells and Chondrogenesis

### 2.1. Adult Stem Cells

Stem cells are mainly classified into two kinds of cells: somatic stem cells and pluripotent stem cells. Somatic stem cells reside in various tissues or organs and have shown multipotency to regenerate damaged tissues [10]. MSCs have been studied intensively in the field of orthopedic research [11]. These cells are characterized as plastic-adherent, fibroblastic spindle-shaped, multipotent stem cells and can differentiate into bone, cartilage and adipose tissue [12]. Also, MSCs are distinguished from hematopoietic cells by being negative for hematopoietic markers such as CD14, CD34 and CD45, but expressing CD29, CD44, CD73 (SH3 and 4), CD90, CD105 (SH2) and CD166 [12,13]. During *in vitro* culture conditions, MSCs are known to change their surface marker expression [14]. MSCs have been isolated from various tissues, such as bone marrow, adipose, synovial tissue, muscle and periosteum [15]. These cell populations are heterogeneous and not clonal populations [14], and MSCs derived from various tissues tend to differ in their expansion capacity and differentiation ability to chondrocytes [16] (Table 1).

Fetal bovine serum (FBS) is widely added to culture medium to expand the populations [17]. However, the potential risk of zoonotic infection or immunogenic reaction is an ever-present danger and a drawback. To reduce these risks, the use of serum free MSC culture media has been developed [18,19]. 

#### 2.1.1. Bone Marrow-derived MSCs (BMMSCs)

In the 1960s, the evidence that bone marrow (BM) includes mesenchymal cells that can generate connective tissue-forming cells was provided by the pioneering work of Friedenstein [20]. A number of investigators extended these observations and confirmed that the cells identified by Frirdenstein were multipotent and could differentiate into osteoblasts, chondrocytes and adipocytes [17,21,22,23,24]. In 1999, Pittenger *et al.* demonstrated that individual human MSCs, which form colonies during their expansion, could retain their multilineage potential [12].The standard methods for the isolation of BMMSCs is density gradient centrifugation method [17]. Using this method, nucleated cells are separated from nonnucleated red blood cells, and thereafter, MSCs are allowed to attach to a plastic culture dish [24]. It is noteworthy that BMMSCs are most widely studied to induce chondrogenesis in three-dimensional cultures. To date, the most promising growth factors for chondrogenesis of BMMSCs are TGF-β superfamily, such as TGF-β1, -β2 and -β3, and members of the BMP family, such as BMP-2, -6 or -7 [12,25,26,27,28,29,30,31,32,33,34]. While TGF-β1 was first used to enhance chondrogenesis [25,26], Barry *et al.* reported that the presence of TGF-β2 or -β3 could also induce chondrogenic differentiation [28]. When they were used in combination of BMP-2 or -6 with TGF-β3, higher collagen II expression was observed than using a single growth factor [31,32]. Although BMMSCs are widely used clinically as a stem cell source [35,36], aspiration of BM is an invasive and painful procedure, often requiring anesthesia and often with attendant morbidity [37].

#### 2.1.2. Adipose Tissue-Derived MSCs (ATMSCs)

In 2001, Zuk *et al.* identified ATMSCs from lipoaspirates, which have multilineage potential to differentiate into adipogenic, chondrogenic, myogenic and osteogenic cells [38]. Following studies also showed the multipotentiality of ATMSCs [39,40]. However, recent studies demonstrated that ATMSCs do not produce results comparable with those of BMMSCs when treated with a variety of growth factors, including TGF-β1, -β2, -β3, BMP-2, -6, -7 or IGF-1 [38,41,42,43,44]. While a combination of BMP-2 and TGF-β1 [45,46] or a combination of BMP-7 and TGF-β2 [44] amplified the chondrogenic potential by more than a single factor alone, combinations of BMP-2, -4 or -7 with TGF-β3 did not show synergetic effects [47]. Moreover, several reports showed that the chondrogenic potential of ATMSCs is not as extensive as that of BMSCs [33,45,48,49]. Despite their inferior chondrogenic potential, interest has increased in the use of ATMSCs, because they are relatively abundant and harvesting techniques of fat tissue might be less invasive than that of BM [33].

**Table 1:** *In vitro* chondrogenesis using adult human stem cells.

Tissue Source	Isolation Method	Culture System	Growth Factors
Bone Marrow [26]	Precoll	Pellet	TGF-β1
Bone Marrow [12,27]	Precoll	Pellet	TGF-β3
Bone Marrow [28]	Precoll	Pellet	TGF-β1, 2, 3
Bone Marrow [29]	Ficoll→CD105(+)cells	Alginate beads	BMP-2, GDF-2
Bone Marrow [30,34]	Ficoll	Pellet	TGF-β3, BMP-6
Bone Marrow [31]	Precoll	Pellet	TGF-β3, BMP-2
Bone Marrow [32]	N/A	Pellet	TGF-β3, BMP-2, IGF-1
Adipose [38]	Collagenase→Attached cells	Micromass	TGF-β1
Adipose [41]	Collagenase→Attached cells	Alginate Beads	TGF-β1
Adipose [42]	Collagenase→Attached cells	Pellet	TGF-β2, IGF-1
Adipose [43]	Collagenase→Attached cells	Alginate Beads	TGF-β1, 3, BMP-6, IGF-1
Adipose [49]	Collagenase→Attached cells	Pellet	TGF-β3
Adipose [44]	Collagenase→Attached cells	Pellet	TGF-β2, BMP-2, 6, 7
Syovium [50]	Collagenase→Attached cells	Micromass	TGF-β1
Syovium [16]	Collagenase→Attached cells	Pellet, collagen gel	TGF-β3, BMP-2
Synovium [55]	Collagenase→Attached cells	Pellet	TGF-β3, BMP-2, IGF-1, FGF-2, Retinoic Acid
Periosteum [50]	Collagenase→Attached cells	Micromass	TGF-β1, BMP2, 4, 7, GDF-5
Periosteum [16]	Collagenase→Attached cells	Pellet	TGF-β3, BMP-2
Muscle [63]	Trypsin→Attached cells	Pellet	TGF-β1
Muscle [16]	Collagenase→Attached cells	Pellet	TGF-β3, BMP-2
Muscle [64]	CD56+34+144+ cells	Pellet	TGF-β3, BMP-4
Traumatized Muscle [65]	Collagenase→Attached cells	Pellet	TGF-β3

#### 2.1.3. Synovium-Derived MSCs (SMSCs)

MSCs from human synovial membrane tissue, known as synovial-derived MSCs, were successfully isolated by De Bari *et al.* in 2001 [50]. Synovial membrane contains two types of cells: macrophage-like cells and fibroblast-like cells; the fibroblast-like cells are believed to be the source of MSCs [51]. Chondrogenesis from SMSCs has been reported by using growth factors such as TGF-β1, -β3 and BMP-2, -7, similar to other adult stem cells [16,52,53,54]. And combination of BMP-2 and TGF-β3 showed higher chondrogenic potential [55]. The fact that both synovium and cartilage are proven to originate from a common pool of progenitor cells [56] suggests that SMSCs may already possess a strong bias toward the production of cartilage tissue. In fact, several studies showed that SMSCs have greater chondrogenic potential than BMMSCs [16,51,57]. Also, it has been reported that multipotent capacity of SMSCs is not influenced by donor age or cell passages, and SMSCs have less senescence and great proliferation ability [16,50]. Clinically, arthroscopic surgery is necessary to obtain synovium, but the procedure is less invasive than harvesting BM. Thus, SMSCs could be an attractive and readily accessible cell source for cartilage regeneration.

#### 2.1.4. Periosteum-Derived MSCs (PMSCs)

Periosteum is at the boundary between the bone and the surrounding soft tissues and contains multiple cell types that are thought to function as progenitor cells. Nakahara *et al.* reported *in vivo* chondrogenic potential of human PMSCs in 1991 [58]. It was not until 2006 that mesenchymal multipotency of adult human periosteal cells was demonstrated by single-cell lineage analysis [59]. They reported that TGF-β1 induced chondrogenesis consistently, but BMP-2, -4, -7 and GDF-5 did not. In the comparative analysis, the chondrogenic ability of PMSCs by treatment with TGF-β3 and BMP-2 was equal to that of BMMSCs [16]. However, harvesting periosteum is invasive and retains a high probability of donor-site morbidity.

#### 2.1.5. Muscle-Derived Stem Cells (MDSCs)

The existence of progenitor cells in the skeletal muscle has been long postulated for a long time, based on the evidence that bone and cartilage formation was observed within the muscle tissue after implantation of demineralized bone matrix [60,61]. The origin of MDSC is thought to be a subpopulation of endothelial cells or pericytes associated with muscle capillaries [62]. Human MDSCs with multilineage potential were first reported by Mastrogiacomo in 2005 [63]. Treatment with TGF-β1 showed chondrogenic differentiation of human MDSCs [63]. Afterward, several groups reported the chondrogenic differentiation by utilizing growth factors including TGF-β1, -β3 or BMP-2, -4 [16,63,64]. Chondrogenic potential of MDSCs is controversial compared to BMMSCs. Whereas MDSCs from normal muscle showed lower chondrogenic potential compared to other MSCs [16], traumatized muscle derived MSCs exhibited similar phenotype and chondrogenic potential [65].

### 2.2. Pluripotent Stem Cells

The heterogeneity of MSC populations isolated from different tissues makes it difficult to determine the most appropriate tissue source for MSCs [66]. Somatic stem cells have limited *in vitro* proliferative potential, and their proliferative capacity and synthetic capacity declines with age [67,68], which restricts the use of MSCs in clinical application. On the other hand, pluripotent stem cells show unlimited proliferative capacity and are attractive for researchers as a better source of stem cells for tissue regeneration [2,66] (Table 2).

**Table 2:** *In vitro* chondrogenesis using human ES and iPScells.

Tissue Source (Cell line )	MSC differentiation Method	Culture System	Growth Factors
ES cell (H9) [78]	EB 5days→EB digestion & plating	Co-culture with chondrocyte	None
ES cell (BG02) [87]	EB 10days→EB digestion & plating	Pellet, PEDGA hydrogel	TGF-β1, BMP-2
ES cell (BG02) [79]	ES colony (cultured with chondrocyte ) derived cells	Pellet, PEDGA hydrogel	TGF-β1
ES cell (H1, H9) [115]	EB 10days→ EB derived cell with, w/o EB digestion	Micromass	BMP-2
ES cell (BG01, 02) [84]	EB 5days→ EB derived cell w/o EB digestion	Self assembly	TGF-β1
ES cell (BG01V) [116]	EB 21days →EB dissociation	Self assembly	TGF-β1, 3, BMP-2, IGF-1
ES cell (H9) [85]	EB 5days→ EB digestion & platingES colony digestion & plating	Micromass	TGF-β1, SB431542
ES cell (H9) [75]	ES colony digestion & plating	Pellet	TGF-β1, BMP-7
ES cell (SA167, AS034, AS034.1) [80]	ES colony (cultured with chondrocyte ) derived cells	Pellet, Agarose gel	TGF-β3
ES cell (BG01V) [88]	EB 21days →EB digestion & self assembly	Self assembly in Agarose gel	TGF-β1
ES cell (H9) [83]	EB 5days →EB micromass 21days→digestion & plating	Pellet	TGF-β1, BMP-2
ES cell (H9) [77]	EB 5days→ EB digestion & platingES colony digestion & plating	Pellet	TGF-β1, BMP-2
ES cell (H9, BG01V) [117]	EB 7, 21, 42days→ EB digestion & self assembly	Self assembly in Agarose gel	None
ES cell (H9) [81]	EB 21days	EB in Agarose gel co-culture with chondrocyte	TGF-β1, BMP-2, 4, 6, PDGFbb, Shh
ES cell (H9) [86]	EB 21days in normoxia / hypoxia	Agarose gel	None
ES cell (H9) [118]	Co-culture with mouse stoma cell followed by CD34+/CD73- sorting	Pellet	TGF-β1
ES cell (HUES3, ZJUhES-1) [119]	ES colony dissociation with Y-27632 (ROCK inhibitor)	Monolayer	TGF-β3
ES cell (H9) [82]	EB 5days →EB micromass 21days→digestion & plating	Pellet, hyaluronan hydrogel	TGF-β1, BMP-2, 7, GDF-5, IGF-1
ES cell (HUES1,7,8) [76]	Direct differentiation from ES colony	Monolayer	Wnt3a, ActivinA, FGF-2, BMP-4, Follostatin, GDF-5, Neurotrophin-4
iPS (fibroblast) [120]	EB 7days→EB derived cells w/o EB digestion	Pellet	TGF-β3
iPS (HDFa-YK26) [114]	iPS colony dissociation with Y-27632 (ROCK inhibitor)	Pellet	TGF-β1
iPS (OA chondrocyte) [2]	OA chondrocyte derived iPS transfected with TGF-β1	Co- culture with normal articular chondrocyte	None
iPS (OA synovial cell) [66]	EB 5days→EB derived cells with EB digestion	Pellet, Agarose gel	BMP-2

#### 2.2.1. Embryonic Stem (ES) Cells

The most well-known pluripotent stem cells are ES cells first reported in the mouse in 1981 and in humans in 1998 [69,70,71]. ES cells are derived from the inner cell mass of a blastocyst. The ability of ES cells to form cartilage is well known, because they can form teratoma containing cartilage when they are transplanted into nude mice [72]. However, the *in vitro* chondrogenesis condition is yet to be determined. As the first step towards differentiation, most ES cell culture protocols follow embryoid bodies (EBs) formation [72]. EBs are free-floating aggregates of ES cells and can differentiate at random into lineages of all three germ layers—endoderm, mesoderm and ectoderm. These EBs, or single cells derived from these EBs, are used in the subsequent differentiation steps. In the first report of *in vitro* chondrogenesis from mouse ES cells, an EB culture system was used. The cartilage like regions displayed various stages of chondrogenesis [73]. Though EB method is convenient for inducing differentiation by the interaction of the cells within the EBs, this method possesses certain limitations. First, it is difficult to dissect the differentiation mechanisms by using single cells. In addition, it is difficult to control the size or number of EBs [74]. Meanwhile, chondrogenesis without EB formation has been reported using co-culture methods or several combinations of growth factors [75,76,77]. Vats *et al.* first reported chondrogenesis of human ES cells using a co-culture method with articular chondrocytes [78]. Co-culture methods were also reported in presence of TGF-β1 or -β3 [79,80]. However, the effects of growth factors are hardly analyzed in the co-culture method. Several growth factors, such as, FGFs, IGF, PDGF and Wnt, have been used for ES cell chondrogenesis without co-culture [72,81,82]. As studied in adult stem cell chondrogenesis, TGF-β1 and -β3 play main roles in ES cell chondrogenesis. BMP-2, -4, -6 and -7 also have been used [76,77,82], and combinations of TGF-β1 and BMPs enhanced the chondrogenic differentiation [75,81,83]. However, these are more complex, as the observed results are dependent on the state of differentiation of the progenitor/stem cells. Although TGF-β1 increased chondrogenesis in differentiated ES derived cells [79,80,84], it inhibited chondrogenesis of undifferentiated EBs [85]. Optimizing culture environment is another approach for ES cell chondrogenesis; a hypoxia condition could enhance chondrogenesis [86], and the culture of EB derived cells in the gel could mimic physiological condition for chondrogenesis [81,87,88]. Although ES cells are an attractive cell source for regenerative medicine, establishment of ES cell lines involves embryo destruction and is overshadowed by ethical concerns for clinical application.

#### 2.2.2. Induced Pluripotent Stem (iPS) Cells

The truly exciting work in 2006 by Takahashi and Yamanaka selected 24 candidate genes that are highly expressed in ES cells. They introduced candidate genes into mouse fibroblasts by retrovirus-mediated transfections and then discovered a minimal combination of four factors (Oct3/4, Sox2, c-Myc and Klf4) could induce the cells to have similar properties as the ES cells. These cells have been christened as induced pluripotent stem (iPS) cells [89]. In 2007, generation of human iPS cells was reported using the same factors [90] or a set of four factors (Oct4, Sox2, Nanog, Lin28) [91]. In addition to mouse and human iPS cells, iPS cells from other species, such as monkey, rat, pig and equine, have already been established [92,93,94,95]. Important features of iPS cells are their unlimited proliferation *in vitro* , while maintaining their pluripotency and their ability of being induced by patient-specific cells [96]. Thus, iPS cells can supply disease or patient specific stem cells for transplantation therapy, drug discovery and oncology or disease pathogenesis research with minimal ethical concerns as compared to ES cells. The original iPS cells exhibit numerous transgene integrations due to retrovirus transfection and tumorigenic risk due to use of c-Myc. To reduce these risks, establishment of iPS cells without c-Myc [97] or using lentivirus, adenovirus or proteins [98,99,100] with a combination of small molecules, which enhance a reprogramming efficiency [101,102], have been reported. Continuous progress in stem cell research has been accomplished by their culture technique. ES cells and iPS cells used to be maintained on mouse-derived feeder cells with animal-derived serum medium. Although the feeder cells are inactivated via radiation or mitomycin, the potential risk of contamination of active mouse fibroblast or zoonotic infection cannot be denied. To avoid these risks, feeder cell- and serum-free culture were established for ES cells [103,104] and applied to iPS cell culture [105,106]. Thus, these technical refinements might lead to safer and more efficient clinical use of iPS cells. 

Treatments of blood, neural and cardiovascular disease models with iPS cell transplantation have already been reported [107,108,109,110]. Compared to these fields, the research for AC regeneration using iPS cells has just begun. Teramura *et al.* reported mouse iPS-EB derived cells expressed surface markers similar to MSCs. These cells could differentiate toward cartilage using TGFβ-3 and BMP-2 [111]. Treatment of EBs with all trans-retinoic acid followed by TGFβ-3 and BMP-2 could also induce chondrogenesis [112]. Regarding human iPS cells, neural stem-cell-derived iPS cell showed chondrogenic potential after EB formation [113]. While in these reports the EB formation method was used, Liu *et al.* reported MSC derivation and chondrogenesis from human iPS cells without EB formation, culturing on thin type I collagen coated plates in presence of ROCK inhibitor Y-27632 [114]. In terms of disease-specific iPS cells, human OA chondrocyte-derived iPS cells have been established. They showed chondrogenic potential using EB formation or co-culture with chondrocytes [2,66] that demonstrated a potential as an alternative cell source for degenerative disease treatment.

## 3. Conclusions

Chondrogenesis using different sources of stem cells has been investigated for several years. However, there are still lingering questions and challenges. Tissue specific MSCs are widely studied; however, their reactions to growth factors vary from tissue to tissue. Even though adult stem cells are isolated from the same tissue, their proliferative rate or differentiation potential vary from patient to patient. Thus, there is still uncertainty about the MSC characteristics. Since these adult stem cells are heterogeneous populations, several surface markers can be used to sort these populations. Standardizing cell isolation and sorting might be useful to analyze the variation between tissue specific stem cells or between patients. ES cells are more proliferative compared to adult stem cells. However, their high proliferative rates have potential oncogenic concerns after transplantation into the recipients. In conclusion, the analysis and optimization of morphogens, such as BMPs and growth factors at each differentiation step, are key to the successful AC regeneration using stem cells.
